# Rapid loss of maternal immunity and increase in environmentally mediated antibody generation in urban gulls

**DOI:** 10.1038/s41598-024-54796-1

**Published:** 2024-02-22

**Authors:** Juliet S. Lamb, Jérémy Tornos, Mathilde Lejeune, Thierry Boulinier

**Affiliations:** 1 Centre d’Écologie Fonctionnelle et Évolutive (CEFE), UMR CNRS 5175, University of Montpellier, EPHE, University Paul Valéry Montpellier 3, IRD, Montpellier, France; 2 Present Address: The Nature Conservancy, Cold Spring Harbor, NY USA

**Keywords:** Conservation biology, Immunology, Ecological epidemiology

## Abstract

Monitoring pathogen circulation in wildlife sentinel populations can help to understand and predict the spread of disease at the wildlife-livestock-human interface. Immobile young provide a useful target population for disease surveillance, since they can be easily captured for sampling and their levels of antibodies against infectious agents can provide an index of localized circulation. However, early-life immune responses include both maternally-derived antibodies and antibodies resulting from exposure to pathogens, and disentangling these two processes requires understanding their individual dynamics. We conducted an egg-swapping experiment in an urban-nesting sentinel seabird, the yellow-legged gull, and measured antibody levels against three pathogens of interest (avian influenza virus AIV, *Toxoplasma gondii* TOX, and infectious bronchitis virus IBV) across various life stages, throughout chick growth, and between nestlings raised by biological or non-biological parents. We found that levels of background circulation differed among pathogens, with AIV antibodies widely present across all life stages, TOX antibodies rarer, and IBV antibodies absent. Antibody titers declined steadily from adult through egg, nestling, and chick stages. For the two circulating pathogens, maternal antibodies declined exponentially after hatching at similar rates, but the rate of linear increase due to environmental exposure was significantly higher in the more prevalent pathogen (AIV). Differences in nestling antibody levels due to parental effects also persisted longer for AIV (25 days, vs. 14 days for TOX). Our results suggest that yellow-legged gulls can be a useful sentinel population of locally transmitted infectious agents, provided that chicks are sampled at ages when environmental exposure outweighs maternal effects.

## Introduction

Wild animal populations can be a valuable surveillance target for monitoring the prevalence and spread of zoonotic disease among wildlife, livestock, and humans^1^. Populations of wild or captive animals with known sensitivity or exposure to infectious agents represent particularly useful targets for monitoring pathogen dynamics^2^. These target populations, or sentinels, can be used for a variety of purposes including measuring changes in circulation of existing pathogens, identifying emerging threats, and predicting the epidemiological consequences of environmental change^3^. However, epidemiological dynamics may vary widely within sentinel populations depending on intrinsic factors including age, condition, breeding status, and social behavior^4^, as well as external factors such as environment, climate conditions, and interspecific interactions^5^. Such variation can in turn affect inferences regarding disease circulation and projected future trends obtained from sentinels, as well as resulting inferences about the broader system. Understanding sources of heterogeneity in epidemiological and immunological parameters is therefore key to choosing appropriate metrics and establishing robust sampling and monitoring schemes for pathogens of interest in sentinel populations.

Antibody seroprevalence is an important tool for understanding pathogen circulation, as it reflects both past and current exposure to disease^6–8^. Antibody testing can also be repeated on the same individuals across multiple occasions to measure infection probability over time^9–11^, making it especially useful for long-term monitoring of exposure rates in sentinel populations. Individuals acquire antibodies via two distinct pathways: maternal transfer, in which antibodies are passively transmitted from mother to offspring during gestation and/or after birth^12–14^, and after external exposure, in which individuals are infected by pathogens and mount an immune response^15^. Maternal antibodies can be transferred from parent to offspring years after the parent was exposed to infectious agents and, once transferred, decline as time passes after birth^16,17^. In contrast, antibodies produced endogenously by an organism reflect the history of exposure of an individual and generally increase over time as an individual accumulates exposure opportunities^10^. Maternal antibody level in the egg yolk is proportional to the level circulating in the female at the time of egg production^18,19^. Maternal antibody persistence after hatching in birds is largely influenced by the amount of antibodies transferred through the egg yolk^16^ and has been known until recently to last only 2–3 weeks after hatching in birds^20^. However, the persistence of maternal antibodies may be longer in some species, potentially as a function of different life history strategies^21,22^, which complicates attribution of antibody titers to intrinsic or extrinsic conditions. Although maternal antibody dynamics have been studied in humans and livestock, they remain relatively unknown in many wild animal populations^14^. Thus, to interpret the external factors driving antibody levels in early life, it is necessary to first understand the dynamics of maternal antibody persistence and differentiate them from those of direct exposure.

Urban wildlife represent an important and increasing interface for disease circulation between animal and human populations^23^. In particular, coastal marine birds such as gulls (Family: Laridae) are highly mobile, interact regularly with human activities, and can contribute to the spread of pathogens among human population centers, making them promising sentinels for detecting and monitoring the circulation of zoonotic pathogens in and around human population centers^24^. Since gulls spend much of the annual cycle dispersed, concentrating in breeding areas during only a few months of the year, antibody seroprevalence offers a longer-term picture of infection dynamics than active infection rates in these species. However, given the wide-ranging movements of marine birds, it can be difficult to pinpoint how and when gulls are exposed to pathogens in the environment. An option for conducting localized surveillance is to focus on nest-bound chicks rather than adults. Nestlings of gulls and other marine birds are altricial and generally spend the first weeks to months of their lives flightless, during which time they are fed by their parents on prey items captured near the colony site. Disease exposure in nestlings is thus reflective of conditions in and around the colony site, making them useful indicators of local conditions and potential sentinels of the circulation of pathogenic agents in coastal systems.

To evaluate the relative contributions of maternal antibodies and exposure to pathogens during development in a seabird sentinel, we studied the dynamics of immune responses to multiple pathogens during development in nestlings of yellow-legged gulls (*Larus michahellis*). The yellow-legged gull is widespread in urban areas of the Mediterranean Sea and hosts and spreads a variety of infectious diseases^25–28^, making it a valuable sentinel species for disease dynamics. Yellow-legged gull chicks have previously been proposed as sentinels for pathogen circulation in marine and coastal environments; however, a lack of information on maternal antibody persistence has limited the interpretation of prior results^29^. To help fill this knowledge gap, we experimentally exchanged eggs between nests and repeatedly sampled antibody levels of chicks after hatch. This method, frequently used in avian studies to separately assess parental and post-hatching effects^30,31^, allowed us to evaluate antibody trends in eggs with similar levels of maternal immunity under differing levels of external exposure. Our results provide valuable context for the interpretation of antibody levels in nestlings, with implications for long-term surveillance of pathogen circulation.

## Methods

### Study site

We conducted our study on the islands of Pomègues and Ratonneau (43° 16′ 32″ N, 5° 18′ 24″ E), located in the archipelago of Frioul in the Mediterranean Sea approximately 2.7 km offshore from the city of Marseille, France (pop. 870,000). The two islands are comprised primarily of bare limestone rock with patches of grassy or shrubby vegetation. They are connected by a dyke and function as a single breeding site, which we will collectively refer to as Frioul. Frioul hosts the largest breeding population of yellow-legged gulls in France with approximately 8,000 breeding pairs, which nest on the island between mid-March and late June. Yellow-legged gulls lay clutches of 2–3 eggs in open cup nests located in grassy or rocky substrates. Eggs typically hatch in mid to late April, and nestlings remain at or near the nest sites (i.e., within 5–10 m) for approximately 35–40 days until they become capable of flight. During the breeding season, adults typically forage either inshore at industrial or agricultural sites, or in nearshore waters around the island (J. Lamb, unpublished data).

### Focal pathogens

We chose three infectious agents potentially pathogenic and of zoonotic interest for exploring exposure in nestling gulls: *Toxoplasma gondii* (TOX), avian influenza virus (AIV), and infectious bronchitis virus (IBV).

*T. gondii*, one of the world’s most common parasites, causes the zoonotic infection toxoplasmosis^32^. Although it can be found in a wide variety of host species, *T. gondii* reproduces only in felids, and is thus highly associated with domestic cats and, by extension, human settlements. The parasite can persist in water and enter marine food webs^33^ and has recently been linked to mortality events in several species of nearshore marine mammals^34,35^. Although *T. gondii* does not always cause noticeable symptoms, it is detectable through serological study^36^, and its ubiquity and wide range of intermediate hosts make it a useful monitoring target^23^. Gulls are an intermediate host for *T. gondii* and can become infected through multiple exposure pathways, including consuming water or refuse contaminated with cat feces, consuming infected marine or terrestrial prey, or scavenging carcasses of infected animals^37^. Gulls infected with *T. gondii* can in turn infect the predators and scavengers that consume them, including feral cats. Thus, migratory birds including gulls can play an important role in maintaining and spreading *T. gondii*, as well as introducing the parasite to uninfected wildlife populations even if no feral cats are present^38^. *T. gondii* is known to circulate in the study population, although with high spatial and temporal heterogeneity^28^.

AIV is perhaps one of the best-known avian diseases, as the few zoonotic strains that exist pose significant public health risks^39^. While most strains are asymptomatic in birds, several have caused widespread avian mortality; thus, predicting the prevalence and spread of AIV is both a conservation and a public health concern^40,41^. AIV is most intensively studied in Anatidae (ducks and geese); however, Charadriiformes (including shorebirds and gulls) are also important reservoirs and long-distance carriers of low-pathogenicity avian influenza viruses, which can mutate into highly pathogenic subtypes^42,43^. Pathways for infection are primarily through fecal shedding and airborne transmission, with the latter likely playing a greater role in disease transmission in gulls compared to ducks and geese^43^. Although AIV infections in gulls are most often gull-specific subtypes (H13 or H16) that do not spill over into domestic fowl, all known clades of AIV have been detected in gulls^42^, with multiple detections of highly pathogenic AIV across a variety of gull species^44^. Early-life infection dynamics and maternal immunity can play an important role in temporal dynamics of AIV infection in gull populations^45,46^, especially since immunity may be longer-lasting in gulls than in ducks and geese^43^. Therefore, a general understanding of AIV antibody dynamics in early life would help provide context for monitoring spatiotemporal variation in AIV prevalence and identifying potential hotspots.

Finally, IBV is a highly infectious avian coronavirus of the gammacoronavirus genus^47^. Although not zoonotic, it is passed from wild birds to poultry and can cause significant mortality in commercially important domestic flocks^48^. Therefore, understanding circulation of IBV in wild populations is of economic interest. IBV, like other coronaviruses, evolves rapidly and is readily transmitted among individuals via both fecal and oropharyngeal shedding^47,49^; thus, migratory species that act as reservoirs could play a key role in both maintenance of IBV and evolution of new strains^50^. In wild bird populations, IBV can co-occur with other respiratory illnesses such as AIV, and synergistic or additive effects of co-infection could exacerbate AIV outbreaks^51^. Gulls are known hosts of novel gammacoronaviruses including IBV^52,53^; however, they are rarely tested for IBV or similar pathogens. Evaluating the prevalence of IBV antibodies in wild gulls could thus indicate whether or not these species play a role in IBV circulation and transmission in this system.

### Nest selection and monitoring

All experiments were performed in accordance with relevant guidelines and regulations. Field sampling for this project was conducted under permits from the Préfecture des Bouches-du-Rhône (Arrêté préfectoral n°13-2020-06-22-00), Parc National des Calanques (Avis Conforme No. DI-2020-046), French Ministry of Research (APAFIS #23794_2020071214191592), Ministère de la Transition Écologique et Solidaire (NOR: TREL2002508S/308) and Centre de Recherches sur la Biologie des Populations d'Oiseaux (CRBPO; Project #1094, Permit #19313) and received approval from the CEFE’s Animal Experimentation Ethics Committee. The study is reported in accordance with ARRIVE guidelines.

Between 28 and 30 March 2021, we selected four separate groups of nests containing 10–20 nests each (i.e., 5–10 pairs) as study plots. For each nest in the study plots with at least two eggs, we briefly floated all eggs in the clutch to determine their approximate developmental stage^54^ and paired nests with eggs at similar stages. Antibody levels in yellow-legged gull eggs decline slightly, with lower antibody levels in the third-laid egg compared to the first-laid egg^55^; since it was often difficult to differentiate exact laying order using floating, we thus chose at random which eggs to exchange, while also prioritizing larger and more advanced eggs in three-egg clutches and ensuring that we exchanged eggs that appeared developmentally similar based on floating angles. We marked the shell of egg that remained in each nest with red non-toxic marker, and the shell of the swapped egg with green marker. If nests contained a third egg, we collected the additional egg as an index of baseline (Day 0) antibody levels for the nest. We retained the egg if the float angle was < 20 degrees, indicating that the yolk could be separated from the white, or destroyed the egg if incubation was too advanced. Previous work^56,57^ has shown that manipulations such as egg removal and exchange do not cause nest abandonment in yellow-legged gulls or other closely-related species.

After swapping eggs, we verified all study nests every two days, switching to daily checks once chicks began to hatch around 10 April. We also collected any additional eggs laid post-swapping and found during subsequent nest checks, so that each study nest contained no more than two eggs. At hatch, we banded chicks on the right tibia with unique metal bands for subsequent identification. In some cases, the tibia was too small to safely band the chick immediately upon hatch. For these chicks, we colored the crown of the head with marker corresponding to their status (green = swap, red = stay) and marked the webbing of the foot with the nest number, refreshing the markings as needed until the chick was large enough to safely band.

To help relocate older chicks, we used a modified Bluetooth-enabled transponder (Chipolo ONE; Chipolo d.o.o., Gabrsko, Slovenia) connected to a smartphone. We removed the transmitter from its plastic housing and re-packaged it in heat shrink tube, which we then attached to the scapular feathers and skin of each chick using epoxy^58^. The transmitter would beep when activated by a nearby connected phone, allowing us to relocate chicks more easily after they left the immediate nest area. We found that chicks were detectable within about 5 m; however, since detecting a chick depended on being able to hear the transponder signal, detection distance depended on ambient conditions and background noise.

### Sample collection

Beginning between days 5–8, we collected 0.5 mL of blood from the brachial vein of each study chick using a syringe and 26G needle. We then repeated serological sampling approximately every 5 days until chicks fledged or disappeared. At each sample collection, we also recorded the mass of the chick using a 2500 g spring scale (Pesola AG, Schindellegi, Switzerland) and measured its head length from skull to bill tip with calipers.

For comparison to samples collected from chicks, we also sampled 30 adult gulls captured on nests as part of a related study between 15 and 24 April 2021. Samples were collected via the same protocol used for chicks. In addition, we opportunistically collected additional samples from nestlings (i.e., chicks ≤ 7 days of age) from 1 April to 18 May 2022 to supplement data on this age class. As these chicks were not part of the egg-swapping experiment, we used them only to develop antibody decay curves and not in sibling comparisons. We captured chicks 1–3 times during sequential visits. Since hatching dates for 2022 chicks were not known, we regressed age (days since hatch) against skull length (head plus bill) and mass for all known-age chicks captured in 2021 and using the resulting linear relationship to estimate chick age from skull length (Fig. S1).

We stored samples in a cooler and centrifuged them upon returning from the colony (i.e., 3–5 h after collection). We then pipetted plasma samples into separate tubes and froze both plasma and red blood cells until beginning lab analyses. For all eggs collected from study nests, we separated egg yolks from egg whites (albumin). We diluted 0.3 µL aliquots of each yolk sample 1:5 in wash buffer, for a total sample volume of 1.5 mL, and stored diluted samples frozen until beginning lab analyses.

### Antibody detection and quantification of antibody titres

We used commercially-available enzyme-linked immunosorbent assay (ELISA) kits to test for TOX, AIV, and IBV antibodies in plasma and egg yolk samples (ID Screen® Avian Toxoplasmosis Indirect, reference no. TOXOS-MS-2P; ID Screen® Influenza A Antibody Competition Multi-species, reference no. FLUACA-5P; and ID Screen® Infectious Bronchitis Indirect, reference no. IBVARSV2-5P; Innovative Diagnostics SARL, Grabels, France). Although these analyses do not provide specific information on strains or subtypes, they offer general information on immune responses and have been successfully used to evaluate antibodies against the target pathogens in wild gull populations in multiple prior studies (e.g.,^28,36,59^). Diluted egg yolks were analyzed following methods previously developed and validated for eggs of the same species^28^. All plasma samples were diluted and analyzed according to kit instructions. For each tray, we included four controls (two positive, two negative) and four standards chosen from our samples representing a range of antibody concentrations. We measured final antibody titres by reading absorbance at 450 nm on a microplate reader (Tecan Infinite® 200 Pro; Tecan Group Ltd., Mannendorf, Switzerland) and correcting raw absorbance values using controls and standard curves. To account for inter-assay variation, we standardized antibody titres by subtracting the average value of the negative controls in the same tray and dividing by the difference between the averages of the positive and negative controls. We also calculated the intra- and inter-assay coefficients of variation (CVs) for each antibody. Intra-assay variation was below 10% in all cases (AIV: 2.9%, TOX: 1.8%, IBV: 1.1%) and was smaller than inter-assay variation, which was nevertheless below 15% (AIV: 9.2%, TOX: 10.6%, IBV: 6.8%). Since early results did not show detectable levels of IBV antibodies in either adult or chick plasma, we did not measure IBV antibody levels in eggs. We also did not measure IBV or TOX antibodies for nestlings sampled in 2022.

### Statistical analysis

To model the processes of maternal antibody decay and external exposure, we fit the sequential values from each individual with a function combining a decay process simulating the loss of intrinsic antibodies and a growth process representing the rate of extrinsic pathogen exposure and antibody generation. Previous studies^16,17,22^ have shown that maternal antibodies typically decay exponentially after hatching in birds, so we chose an exponential decay function to represent maternal antibody loss. The form of antibody generation following exposure to an infectious agent is less clear, so we tested two alternatives: a linear growth process (i.e., mounting of the immune response after potential exposure is constant throughout development) and a sigmoidal growth process (i.e., mounting of the immune response after potential exposure is low in early life, increases as chicks become mobile, peaks halfway through development, and slows toward fledging as antibody levels approach an asymptote). Thus, we tested two forms of the relationship between antibody level and chick age:

1 $$T \sim ({\alpha e}^{-\lambda d})+(\beta d)+ \varepsilon$$

and

2 $$T \sim (\alpha {e}^{-\lambda d})+(1+{{e}^{-\beta (d-20)})}^{-\gamma }+ \varepsilon$$

where $T$ is the antibody level (ELISA titre value), $d$ is the number of days since hatch, $\alpha$ is the starting value at Day 0, λ and β are the rates of antibody decay and generation respectively, γ is the asymptote, and $\varepsilon$ is the residual variance. To account for repeated sampling of individuals whose starting values varied, we fit these equations in a non-linear mixed modeling framework, in which the asymptote (i.e., initial maternal antibody level) was allowed to vary by individual. We fit models with a Gaussian distribution and identity link via the nlme package^60^ in R 4.2.0 (R Core Team 2020). To determine which model best represented the observed patterns of antibody generation, we compared Akaike Information Criterion (AIC) values of the two candidate models and, provided model scores were separated by > 2 points, chose the model with the lowest AIC value as the top model^61^. We then conducted fivefold cross validation of the selected model for each pathogen and calculated mean root mean squared error (RMSE;^62^) as a measure of goodness of fit.

To compare chick antibody levels with those of their biological and foster siblings we first interpolated daily antibody titre values for each chick based on the best-fitting non-linear model selected in the previous step. We then assessed the relationship of antibody titres to chick age and sibling values using generalized additive mixed models (GAMMs) fitted via the gamm4 package^63^ in R. We constructed a separate model for each pathogen that included a main effect of chick age, interactive effects of age with biological sibling titre and foster sibling titre, and a random effect of chick identity nested in study plot to account for repeat sampling and spatial autocorrelation. We considered a predictor to be significant if the 95% confidence interval of its coefficient estimate did not overlap zero.

## Results

**Figure 1:**
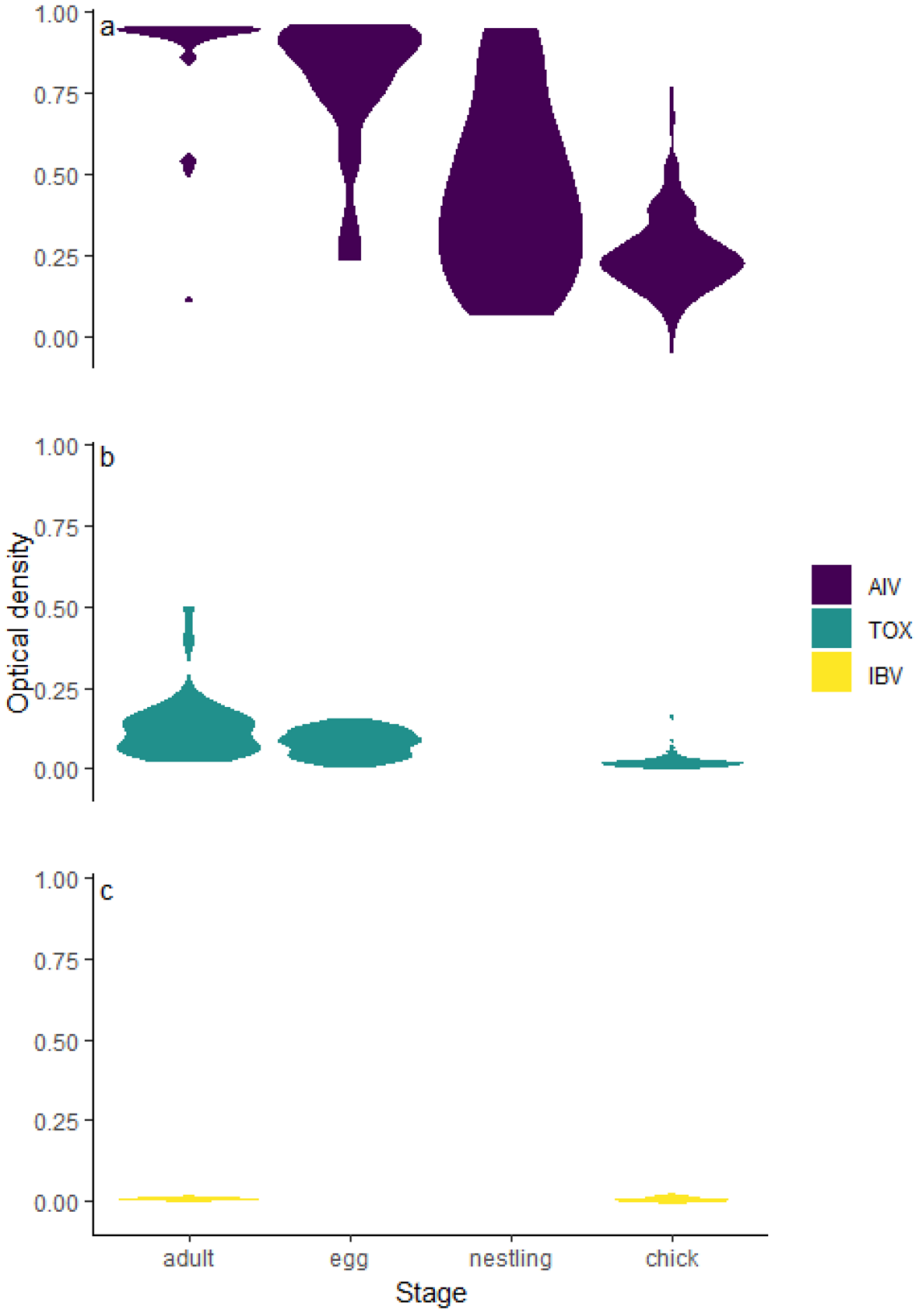
Distribution of antibody levels against (**a**) avian influenza virus (AIV), (**b**) *Toxoplasma gondii* (TOX), and (**c**) infectious bronchitis virus (IBV) in yellow-legged gulls adults (*n* = 90), eggs (*n* = 108), nestlings (≤ 7 days old; *n* = 28) and chicks (> 7 days old; *n* = 114) on Frioul, 2021–2022. Note: we did not test for TOX and IBV antibodies in nestlings, or for IBV antibodies in eggs.

Antibody levels against AIV were high and prevalent across the population (CV = 0.43, Fig. 1A). AIV antibody levels were highest in adults, declining gradually through the egg, nestling, and chick stages. TOX antibodies were generally low but varied among age classes and individuals (CV = 1.18). We found higher antibody levels in adults and eggs than in chicks, and several individuals showed higher antibody levels consistent with recent exposure (Fig. 1B). IBV antibodies were not detected in any of the individuals sampled (Fig. 1C). Antibody levels against AIV detected in eggs (i.e., day 0) were strongly positively correlated with antibody levels detected in the first samples of chicks from the same nest (days 8–15) (coefficient = 1.2 [95% CI = 0.82–1.58], *t*_17_ = 3.20, *p* = 0.005); however, TOX antibody levels at day 0 were not correlated with the first chick samples from the same nest (coefficient = 1.2 [95% CI = -1.72–4.15], *t*_17_ = 0.81, *p* = 0.45).

For both AIV and TOX, the best-fitting models combined an exponential decrease and a linear increase in antibody levels with age (i.e., Eq. 1) (AIV: AIC = -247, vs. -241 for Eq. 2; TOX: AIC = -2431 vs. -2428 for Eq. 2). The selected models were a good fit for the observed data (AIV: RMSE = 0.19 ± 0.04; TOX: RMSE = 0.02 ± 0.02).

**Figure 2:**
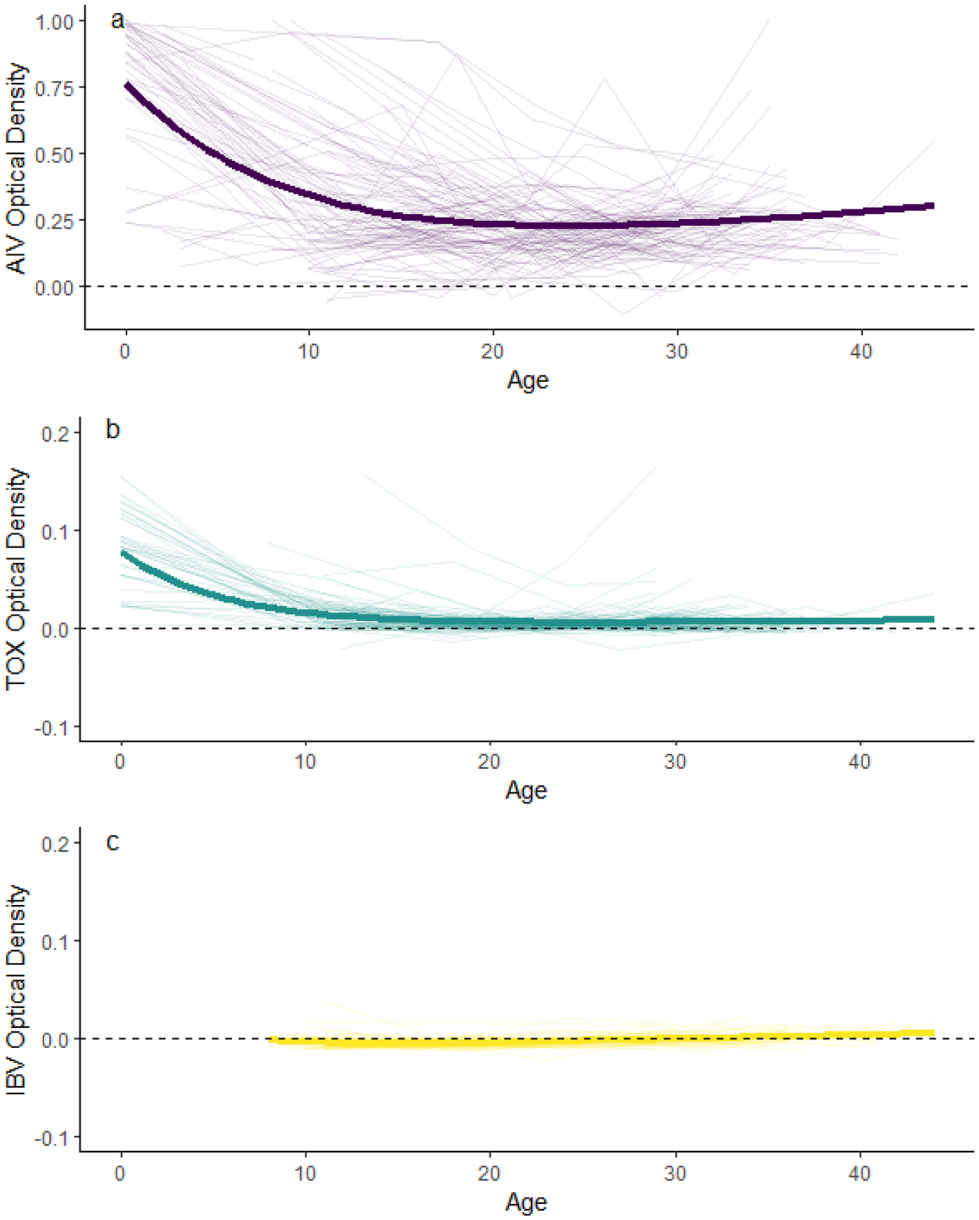
Changes in antibody concentrations from hatching (Day 0, egg stage) through fledging (Day 40) in yellow-legged gull chicks (*n* = 142) on Frioul, 2021–2022: (**a**) avian influenza virus (AIV), (**b**) *Toxoplasma gondii* (TOX), and (**c**) infectious bronchitis virus (IBV). Figure includes individual trajectories in antibody concentrations (light lines), fitted values derived from the best-fitting non-linear model combining exponential decrease in maternal antibodies and linear increase in antibody generation (heavy lines), and zero values (dashed lines).

For AIV, we estimated the starting antibody level (α) at 0.77 (95% CI = 0.09–0.12; *t*_540_ = 31.69, *p* < 0.001), the rate of decay in maternal antibodies (*λ*) at 0.10 (95% CI = 0.09–0.12; *t*_540_ = 13.76, *p* < 0.001), and the daily rate of increase in antibodies (*β*) at 0.007 (95% CI = 0.006–0.008; *t*_540_ = 12.29, *p* < 0.001) (Fig. 2A). For TOX, we estimated the starting antibody level (α) at 0.08 (95% CI = 0.07–0.08; *t*_487_ = 28.29, *p* < 0.001), the rate of decay in maternal antibodies (*λ*) at 0.17 (95% CI = 0.14 –0.21; *t*_487_ = 36.48, *p* < 0.001), and the daily rate of increase in antibodies following external exposure (*β*) at 0.0002 (95% CI = 0.0001–0.0003; *t*_487_ = 17.07, *p* < 0.001) (Fig. 2B). IBV antibody levels remained constant at or near 0 throughout the study period. (Fig. 2C; 95% CI = -0.0001–0.0002, *t*_247_ = 0.04, *p* = 0.67).

**Figure 3:**
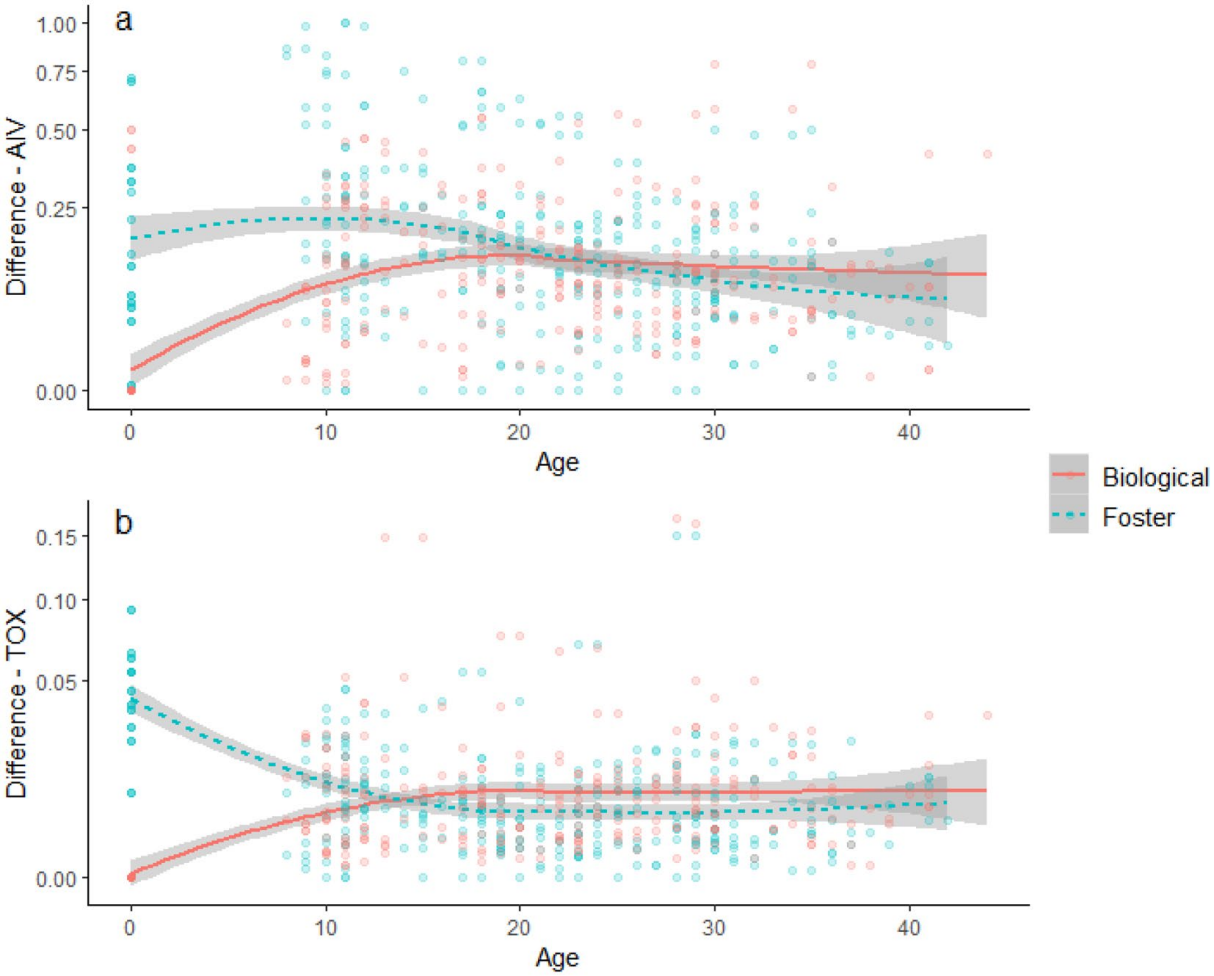
Inter-pair differences in antibodies against (**a**) avian influenza virus (AIV) and (**b**) *Toxoplasma gondii* (TOX) between sets of biological siblings (blue) and foster siblings (pink) for yellow-legged gull chicks (*n* = 670 samples) from Day 0 (hatch) to Day 40 on Frioul, 2021. Dots show daily differences between siblings, and lines show mean differences across the population with 95% confidence intervals (shaded).

Differences in antibody titres between biological siblings increased rapidly during the first 2—3 weeks post-hatch for both AIV and TOX before reaching a plateau (Fig. 3). Differences in AIV antibody titres between foster siblings remained constant through the first 17 days post-hatch and then declined through the rest of the study period, such that biological and foster siblings were indistinguishable by Day 22 (Fig. 3A). In contrast, differences in TOX antibody levels between foster siblings declined after hatch before leveling off (Fig. 3B), and foster siblings were more similar to one another than biological siblings from Day 14 onward.

**Figure 4:**
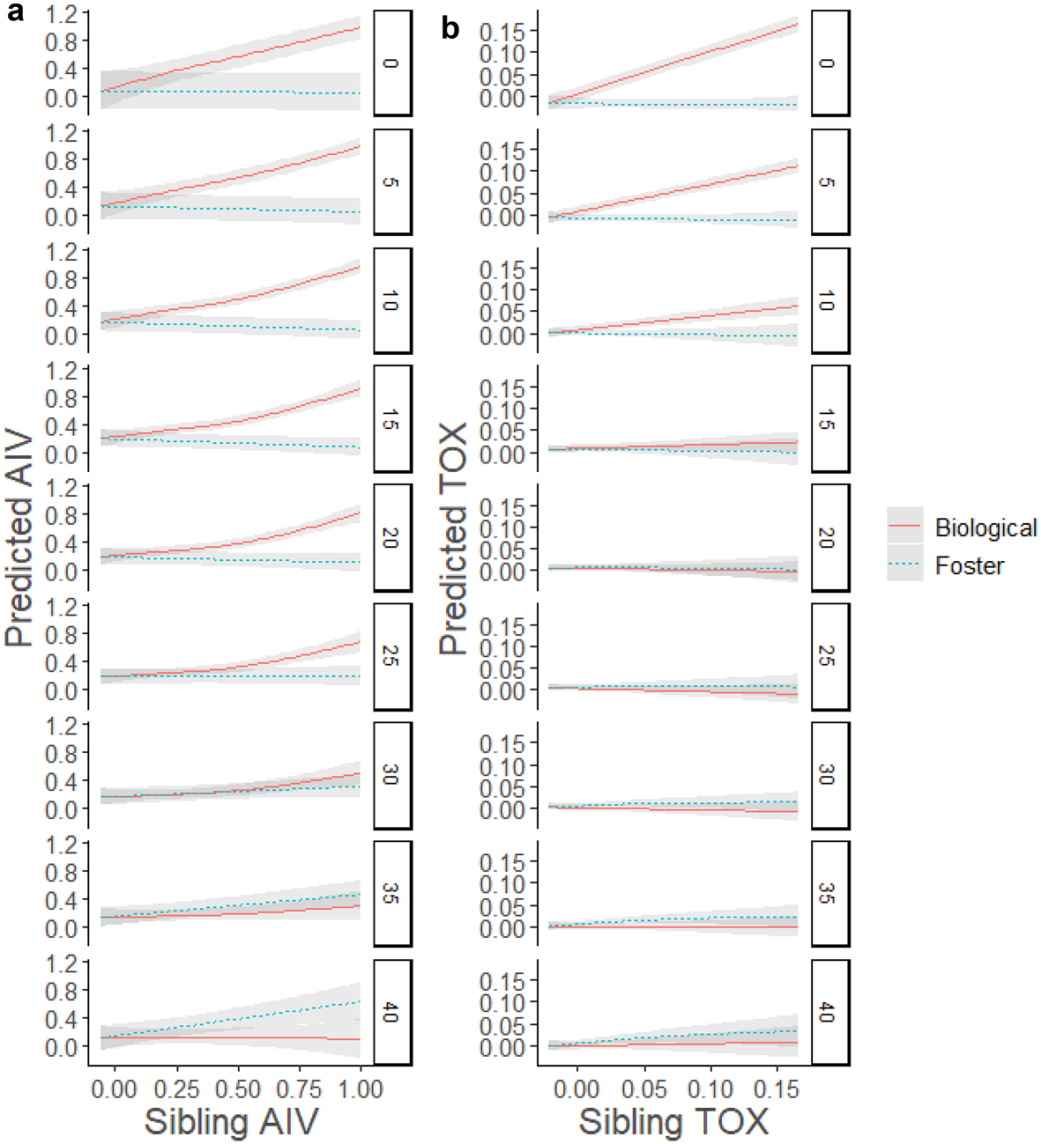
Generalized additive model fits of the relationship between sibling titre and predicted titre for a) avian influenza virus (AIV) and b) *Toxoplasma gondii* (TOX) at 5-day intervals for yellow-legged gulls on Frioul, 2021. Relationships are shown for biological siblings (solid red line) and foster siblings (dotted blue line) with shaded areas indicating 95% confidence intervals. Labels indicate chick age in days from 0 to 40.

The GAMM for predicted AIV titre included a strong interaction of biological sibling titre with age (edf = 6.95, *F* = 11.27, *p* < 0.001) and a weak interaction of foster sibling titre with age (edf = 3.28, *F* = 2.23, *p* = 0.07); the main effect of age was not significant (*p* > 0.1). AIV titres of biological siblings were positively correlated from hatch through day 25, with stronger correlations between siblings with higher AIV titres (Fig. 4A). AIV titres of foster siblings were positively correlated after day 35, albeit with substantial variability (Fig. 4A). The GAMM for predicted TOX titre included a strong interaction of biological sibling titre with age (edf = 5.86, *F* = 41.6, *p* < 0.001), and no effect of age or foster sibling titre–age interaction (*p* > 0.5 for both). There was a strong positive relationship between TOX titres of biological siblings from hatch through day 15, after which they were no longer correlated (Fig. 4B).

## Discussion

Our results highlight the value of sampling across multiple life stages, as well as the need to consider both chick age and background levels of pathogen circulation when interpreting antibody concentrations in nestlings.

The three pathogens we tested differed markedly in their overall prevalence across the study population. Antibody levels against AIV were consistently high in adults, eggs, and chicks, suggesting broad exposure. Antibody levels against TOX were considerably lower across all life stages, although a few individuals showed relatively high concentrations of antibodies; levels also declined markedly from adults to chicks. IBV antibody levels were at or near zero regardless of life stage. These three pathogens thus provide a useful gradient of background levels of exposure and local pathogen circulation, from prevalent, to rare, to absent. Differences in antibody levels among pathogens and individuals were most pronounced in adults, suggesting differences in past history of exposure. However, a drawback to measuring antibody levels in adults is that it can be difficult to pinpoint the timing of exposure^7^, as the duration of antibodies after infection may vary depending on the pathogen in question, the severity of the initial infection, and previous exposure history^64,65^. Antibody levels in eggs showed a similar gradient from AIV (high), TOX (intermediate), to IBV (low), albeit at lower levels than in adults. Antibody levels transferred by mothers to eggs vary with antibody levels in maternal serum^55^, making them subject to similar sources of individual variation as in adult samples, and can also be influenced by factors unrelated to pathogen circulation such as local food availability and maternal quality^55,66^. Antibody levels in chicks, whose exposure history is limited to the breeding period, were lower than in either adults or eggs, with younger nestlings having higher overall antibody levels than older chicks. Although among-individual and among-pathogen variation in antibody concentrations were generally less pronounced in chicks compared to eggs or adults in our study, comparative levels of immune responses to different pathogens appeared similar across all age classes. Thus, despite differences in magnitude, all age classes sampled provided useful indicators of background differences in pathogen exposure across the population.

During the chick stage, maternal antibody levels against avian influenza virus and *T. gondii* declined at similar rates. However, starting levels of AIV antibodies in eggs were approximately eight times higher than TOX antibody levels, resulting in a longer duration of apparent maternal effects inferred from egg swapping (25 vs. 14 days). This confirms previous findings^17^ that the duration of maternal immunity in nestlings depends heavily on starting antibody levels (see also^16^). Our work provides an interesting complement to Ramos et al., in that the previous study compared differing levels of experimental exposure to a single pathogen via vaccination, while ours compared natural exposure to different pathogens in wild-caught birds. This between-pathogen comparison suggests that nestlings could be expected to have longer-lasting immunity to pathogens that are more prevalent in their natal systems, which may provide fitness benefits in the form of increased early-life survival^67^. We did not assess strain-specific immune responses against different AIV subtypes in this study; however, this would be a valuable direction for future work, especially in light of recent outbreaks of high-pathogenicity AIV in seabird colonies worldwide. While early-life immunity could potentially play an important role in buffering against future outbreaks, immunity is likely to be highly subtype-specific^45,46^.

Although rates of maternal antibody decay were similar between AIV and TOX, rates of antibody generation to the two pathogens differed by several orders of magnitude. While maternal antibodies can be influenced by exposure of adults to pathogens away from the breeding area, antibody generation rates in chicks reflect only localized pathogen circulation. High rates of immune response to AIV in chicks indicate that AIV circulates widely in this population, which is also supported by the fact that almost all sampled adults were seropositive to AIV antibodies. We did not measure active infection rates; however, since AIV can be readily transmitted via either respiratory or fecal–oral pathways, chicks in dense breeding colonies are likely to be exposed to the virus even if only a small percentage of adults are infected. In contrast, the low rate of generation of TOX antibodies in chicks suggests limited exposure to *T. gondii* in the local system around the breeding colony during the study period. Compared to AIV, TOX presents more limited opportunities for transmission, requiring direct contact with cat feces in water or soil or consumption of infected carcasses or prey items. Feral cats are present on Frioul; however, their use of gull nesting areas is limited, and they may be isolated from mainland sources of *T. gondii*. An analysis of eggs collected from multiple Mediterranean colonies of yellow-legged gulls^28^ found strong interannual and spatial heterogeneity in *T. gondii* antibody levels, with antibodies detected in anywhere from 5 to 45% of eggs from Frioul, depending on the year. This suggests that adult exposure and infection rates, and thus maternal antibody transfer, may vary among years. However, this variation is not necessarily linked to conditions at the breeding colony, since adults may attend distant wintering sites. We found higher overall antibody levels and greater inter-individual variation in adults and eggs compared to chicks, which supports the idea that exposure may be occurring outside the local area. Multi-annual serological sampling of nestlings ≥ 15 days old would help to determine whether local-scale *T. gondii* circulation also varies interannually, or whether adult exposure is more closely linked to conditions at wintering sites or flexible migratory strategies.

By comparing cross-fostered nestlings, we confirmed that maternal effects were dominant over external exposure to pathogens for the first three weeks of chick growth for AIV and the first two weeks for TOX. After this point, antibody titres in foster siblings were slightly more similar to one another than biological siblings for AIV, but not for TOX. This suggests that nest-specific differences in external exposure are relatively minimal across the breeding colony and may vary among pathogens with different transmission routes. AIV can be transmitted through both respiratory and fecal–oral pathways; thus, infection probability depends on proximity to infected birds, which is likely to vary among but not within nests. Since chicks are most likely to be exposed to TOX by consuming contaminated food or water, similar exposure rates among nests could result from relatively uniform adult foraging behavior during the breeding season. Indeed, tracking data from Frioul confirm that adults congregate at similar mainland foraging areas and show limited individual specialization (Lamb et al., in prep). This contrasts with numerous examples from other gull species in which adults specialize on specific foraging habitats by utilizing relatively more marine or terrestrial areas^68,69^ or targeting different food resources^70–72^. Frioul is located along a highly developed coastline close to a dense urban area, and the abundant and predictable anthropogenic food resources close to the island may limit foraging habitat partitioning and, thus, opportunities for differential pathogen exposure during the breeding season, as previously demonstrated in herring gulls (*Larus argentatus*)^73^. Consequently, increased heterogeneity in TOX antibodies might be expected if anthropogenic food resources were to decline—for example, if management practices further limit access to landfills and waste treatment stations.

Our results provide several practical considerations when using yellow-legged gulls as a sentinel species for pathogen circulation at the wildlife-human interface. Notably, we show that antibody levels offer a tool for comparing prevalence of different pathogens in the system. It is important to note that different life stages provide information at different spatial and temporal scales—adult exposure rates and antibody levels in eggs depend on annual-cycle habitat use by mobile breeders, while exposure rates in nestlings respond to local conditions at or near the breeding site. We also show that nestling antibody rates are primarily driven by maternal immunity for the first several weeks of nestling growth, with the exact duration of maternal immunity depending on the initial levels of antibodies deposited in eggs. Our results suggest that maternal immunity in yellow-legged gull nestlings declines exponentially at similar rates regardless of starting levels, meaning that it could be possible to correct for maternal transfer in younger known-age chicks. However, sampling older chicks (at least two weeks of age for less-common pathogens, or up to four weeks for more prevalent pathogens) provides the most efficient way to assess pathogen circulation in the very local environment. After this point, we found that chicks from different nests showed similar antibody levels, meaning that sampling would not need to account for within-colony variation in exposure. Finally, although we were able to obtain robust estimates of daily increase in exposure by sampling the same chicks multiple times in a season, this required extensive time and repeated disturbance. A single sample could offer a relatively simple and practical alternative for monitoring variation among years or sites, provided that the chicks are sampled relatively close to fledging and are of similar ages across years.

### Supplementary Information

Supplementary Information.

## Data Availability

Data and code used to conduct the analyses in this manuscript are available on Zenodo: http://doi.org/10.5281/zenodo.10260818.
