# Supplementary material for: Rapid loss of maternal immunity and increase in environmentally mediated antibody generation in urban gulls
Source: Sci Rep. 2024 Feb 22;14:4357. doi: 10.1038/s41598-024-54796-1 (PMC10884025; doi:10.1038/s41598-024-54796-1)

Rapid loss of maternal immunity and increase in environmentally mediated antibody generation in urban gulls

Supplementary Information

Juliet S. Lamb, Jérémy Tornos, Mathilde Lejeune, and Thierry Boulinier

Figure S1. **Changes in a) skull length (head + bill) and b) weight with age for yellow-legged gull nestlings captured on Frioul, 2021-2022.** Dotted lines represent regression equations for each parameter. Known-age chicks captured in 2021 were used to derive the regression relationship and are shown in lighter colors. Unknown-age chicks captured in 2022 are shown in darker colors, with age estimates derived from a multiple regression equation combining both parameters.

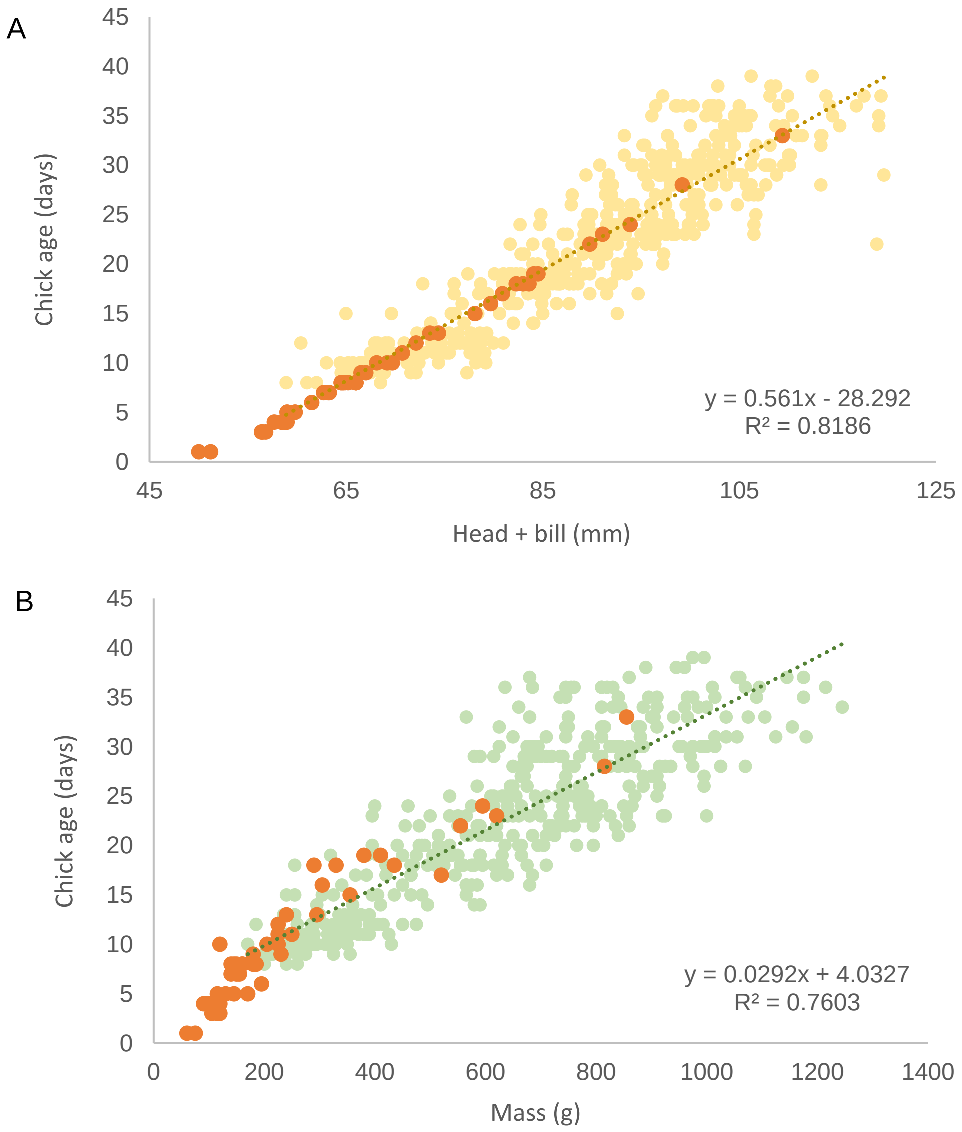

Supplement: Supplementary file 1 — Supplementary Information. [file 41598_2024_54796_MOESM1_ESM.pdf]
